# Farm Animal Welfare Is a Field of Interest in China: A Bibliometric Analysis Based on CiteSpace

**DOI:** 10.3390/ani13193143

**Published:** 2023-10-08

**Authors:** Lihang Cui, Wenjie Tang, Xiaoshang Deng, Bing Jiang

**Affiliations:** 1 College of Economics and Management, Northeast Agricultural University, Harbin 150030, China; b210801002@neau.edu.cn (L.C.); s220801029@neau.edu.cn (W.T.); a08200873@neau.edu.cn (X.D.); 2 Development Research Center of Modern Agriculture, Northeast Agricultural University, Harbin 150030, China

**Keywords:** farm animal welfare, China, research trends, research hotspots, research frontiers, bibliometric analysis, CiteSpace

## Abstract

**Simple Summary:**

Farm animal welfare research conducted in China is relatively less accessed and unknown outside of the country, which might create the impression that farm animal welfare receives limited attention in China. This paper aims to offer a comprehensive review of the existing Chinese farm animal welfare literature. CiteSpace software was employed for the analysis and visualization of various aspects of the literature, including the quantity, species, authors, institutions, journals, and keywords. This process helped reveal the research trends, hotspots, and frontiers in the field. The findings from this bibliometric analysis strongly indicate that farm animal welfare is a subject of considerable interest and research activity in China.

**Abstract:**

Farm animal welfare research conducted in China is not commonly accessed or known outside of China, which may lead to the assumption that farm animal welfare receives relatively little attention in China. Therefore, a bibliometric analysis was conducted on the existing Chinese farm animal welfare literature to provide robust evidence to refute this assumption. A total of 1312 peer-reviewed Chinese studies on farm animal welfare published between March 1992 and June 2023 were retrieved from the Web of Science (WoS) and the China National Knowledge Infrastructure (CNKI) database. CiteSpace software was used to analyze and visualize the number, species, authors, institutions, journals, and keywords of the papers. In China, farm animal welfare research has gone through the processes of an early stage (1992–2001), rapid-growth stage (2002–2007), and mature stage (2008–present), and the scale of research continues to grow. Notably, swine and chickens have received priority attention in this area. A Matthew effect was observed for authors and institutions, with relatively little collaboration among authors and institutions. Most of the papers were published in a small number of journals, with an apparent agglomeration characteristic. The research hotspots, summarized as “feed and diet”, “environmental impacts and control”, “integrated rearing management”, “injury and disease”, “behavior and technologies for behavior monitoring”, “genetic analysis”, “welfare during transport and slaughter”, “welfare-friendly animal product consumption”, “attitudes toward farm animal welfare”, and “healthy breeding”. The keywords “computer vision”, “recognition”, “temperature”, “precision livestock farming”, “laying hen”, and “behavior”, represent the major research frontiers in the field, which could indicate potential areas of significant future research. The findings of the present bibliometric analysis confirm the fact that farm animal welfare is a field of interest in China. Farm animal welfare research in China tends to be pragmatic, with a strong emphasis on enhancing growth and production performance, as well as product quality, rather than solely concentrating on improving farm animal welfare. This paper provides insightful references that researchers can use to identify and understand the current status and future direction of the farm animal welfare field in China.

## 1. Introduction

The demand for the most popular farm animal products (i.e., meat, eggs, and milk) in China has expanded from 45.5 kg per capita in 2013 to 60.5 kg per capita by 2021 [1]. In line with this trend, livestock and poultry production in China has experienced significant growth over the past two decades. The production of main farm animal products in China has increased by 8.85% at an average annual rate of 2.43% from 2003 to 2022 [1]. However, to meet the increasing consumption demand, the production growth has primarily stemmed from the rapid growth of intensive livestock and poultry farming practices [2]. Although the intensification of farm animal production significantly boosts productivity and economic benefits, it inevitably leads to farm animal welfare issues and compromises the sustainability of animal husbandry [3].

Farm animal welfare concerns have often been neglected in China due to the Chinese government’s prioritization of economic development [4]. This fact is demonstrated by the absence of nationwide laws and official controls pertaining to farm animal welfare [5,6]. In particular, when facing the pressure of the rising demand for farm animal product consumption in the country, it is virtually impossible to reduce intensive livestock and poultry farming practices [4]. For example, the previous version of the Chinese national standard “Construction for Intensive Pig Farms”, which was released in 2008, initially stipulated a stocking density of 0.8–1.2 m^2^/pig for growing-finishing pigs. However, in the revised version of 2022, the stocking density was adjusted to 0.5–1.0 m^2^/pig [7]. In view of China’s dominance in the production and exportation of farm animal products, the country is receiving global attention over its farm animal welfare issues [6].

Despite this, there are encouraging signs of positive changes concerning farm animal welfare in China. Several farm animal welfare standards have been established by government departments, social groups, industry associations, and enterprises over the past few years, providing valuable guidance for livestock and poultry production practices. One example is the “Farm Animal Welfare Requirements” released by the International Cooperation Committee of Animal Welfare (ICCAW), which covers various farm animal species, including pigs, beef cattle, mutton sheep, meat-type chickens, laying hens, waterfowl, cashmere goats, and dairy cattle [8]. Moreover, an increasing number of Chinese livestock and poultry enterprises, such as Suzhou OVODAN Foods Co., Ltd. (Suzhou, China), are launching welfare-friendly animal products, particularly eggs and chicken. These products have received certification from organizations such as Humane Farm Animal Care [9]. Furthermore, China is actively, widely, and increasingly participating in the global movement to improve the welfare of farm animals. By the end of 2022, the World Conference on Farm Animal Welfare had been held in China for four consecutive sessions [10]. Driven by food safety considerations, the Chinese public has also shown an increasing level of empathy and concern toward farm animals in recent years [11,12].

Nevertheless, the development of animal welfare in China remains in an early stage, and more practical measures need to be implemented to promote its development [11]. Due to distinctive historical and cultural backgrounds, political systems, basic national conditions, etc., different countries face unique problems and require specific solutions to address farm animal welfare issues. Therefore, farm animal welfare in China is best addressed by using Chinese solutions rather than relying solely on solutions designed by international organizations or dictated by global trends [13]. In many countries with high farm animal welfare standards, reform has been fueled by and based on scientific research in farm animal welfare, particularly in European Union countries [14]. However, the scientific literature on farm animal welfare from China remains relatively scarce when compared to the extensive literature available from Europe, Oceania, and America, where farm animal welfare is highly developed [15]. This has even led to the potentially inaccurate viewpoint that farm animal welfare is of little interest or concern in China [14]. Many worthwhile results and opinions on farm animal welfare in China may also be overlooked by other researchers [15]. For this reason, a systematic review of the existing Chinese farm animal welfare literature is necessary to understand the detailed context of Chinese studies on farm animal welfare.

Very few reviews of Chinese farm animal welfare research have been conducted, with only one systematic review being retrieved. Specifically, a bibliometric analysis was conducted by Sinclair et al. [14] on 854 Chinese-language academic publications related to pig and poultry welfare from Chinese scientific databases between 2008 and 2018. The attention of Chinese academia in the field of farm animal welfare was clarified by identifying the basic characteristics of the publications, including the annual publication count, topic categories, species, age of animals, and production stage [14]. Although several important conclusions were drawn from the study, the current situation and development trend within the field of farm animal welfare research in China remains ambiguous, including aspects such as the publication trends, the contributing authors, institutions and journals, collaborative relationships among authors and institutions, as well as research hotspots and frontiers. Additionally, the study was limited to Chinese-language publications related to pig and chicken species from Chinese scientific databases. Therefore, further research is required to delve deeper into these aspects, address the identified limitations, and provide a more comprehensive understanding of the field.

Therefore, the present study aimed to perform a bibliometric analysis on farm animal welfare research in China. Specifically, publication outputs, the distribution of species, and highly productive authors, institutions, and journals were analyzed. Then, cooperation relationships among authors and institutions were identified. Furthermore, research hotspots and research frontiers were clarified, and future research development directions were predicted. The findings of this study could help readers understand the current status of farm animal welfare research in China and grasp its future direction.

## 2. Materials and Methods

### 2.1. Literature Search Strategy

The literature search for this review utilized the databases of the Web of Science (WoS) and China National Knowledge Infrastructure (CNKI). WoS (https://www.webofscience.com, accessed on 12 June 2023) is widely acknowledged as the most commonly used and widely accepted digital database in scientific research, providing researchers with various types of high-quality publications [16]. Therefore, the WoS core collection was selected as the source of non-Chinese-language literature (i.e., Chinese research conducted in China and published in a foreign language), which is consistent with previous studies [17,18]. CNKI (https://www.cnki.net, accessed on 12 June 2023) is the largest and most authoritative continuously updated database for scientific publications in China [19]. It encompasses various academic resources, such as journals, master’s and doctoral dissertations, conference proceedings, newspapers, yearbooks, eBooks, patents, standards, etc. [20]. Among these types of publications, the literature published in journals were found to be relatively more continuous, sensitive, and directly related to the academic field [21]. Therefore, the Chinese-language literature (i.e., Chinese research conducted in China and published in the Chinese language) for this review was obtained from academic journals within the CNKI database, which is in accordance with previous studies [14,22]. Furthermore, to ensure the quality of the literature, the literature search was conducted on peer-reviewed journals only [23]. Thus, the Science Citation Index Expanded (SCI) and Social Science Citation Index (SSCI) databases within the WoS core collection, as well as the Chinese Core Journals of Peking University, Chinese Social Sciences Citation Index (CSSCI), and Chinese Science Citation Database (CSCD) within the CNKI academic journals database were selected as literature source categories.

The search term for retrieving literature was not restricted to “farm animal welfare” alone since this could have led to potential errors of data omission. This is because “farm animal” is a generic term for livestock and poultry. When referring to certain specific types of livestock or poultry, scholars often prefer to use the name of specific farm animals directly, rather than the term “farm animal”. In the current study, swine, beef cattle, dairy cattle, sheep, goats, broiler chickens, laying hens, ducks, and geese were selected as the major specific species of livestock and poultry for the following reasons: (1) Swine, beef cattle, dairy cattle, sheep, goats, broiler chickens, laying hens, ducks, and geese are considered the most important farm animals in China, representing the largest proportion of the farm animal population and the highest number of animals slaughtered [24]. (2) The exportation and domestic consumption of pork, beef, veal, milk, and lamb, as well as the meat and eggs of chickens, ducks, and geese, ranked highest among farm animal products in China. The welfare of these farm animals is critical since it can have potential impacts not only in China but also in other regions across the world [25]. (3) Welfare-friendly animal products, such as pork, chicken meat, chicken eggs, and milk, have been increasingly and widely promoted in China [26]. (4) Swine, poultry, cattle, and sheep are the key farm animal species for the implementation of the national standard “Welfare on Killing Animals for Disease Control Purposes”, which is the only national standard for farm animal welfare currently enforced in China [27].

Given the above, numerous search experiments were conducted using search terms to ensure the comprehensiveness and accuracy of the literature. Referring to Qiang et al. [28], non-Chinese-language literature was limited to research conducted in China by setting the “Address” to “China”. The final search term for non-Chinese-language literature was set as {(TS = “farm animal welfare” AND AD = “China”) OR [TS = (“swine” OR “pig” OR “sow” OR “boar” OR “piglet” OR “fattening pig”, “cattle” OR “cow” OR “bovine” OR “bull” OR “beef cattle” OR “dairy cattle” OR “dairy cow” OR “calves”, “sheep” OR “goat” OR “lamb”, “chicken” OR “hen” OR “rooster” OR “laying hen” OR “broiler”, “duck”, “goose”, “livestock” OR “poultry” OR “husbandry”) AND TS = (“farm animal welfare” OR “animal welfare” OR “welfare”) AND AD = “China”]}, including plurals. The final search term for Chinese-language literature was set as {主题 = (“动物福利” OR “农场动物福利”) OR 篇关摘 = (“动物福利” OR “农场动物福利”) OR [主题 = (“猪” OR “母猪” OR “公猪” OR “仔猪” OR “育肥猪”, “牛” OR “母牛” OR “公牛” OR “肉牛” OR “奶牛”, “羊” OR “母羊” OR “公羊” OR “绵羊” OR “山羊”, “鸡” OR “母鸡” OR “公鸡” OR “蛋鸡” OR “肉鸡” OR “仔鸡”, “鸭”, “鹅”, “畜牧” OR “家禽” OR “畜禽”) AND 主题 = (“福利” OR “动物福利”)]}. The English translation of the search term for Chinese-language literature is {Subject = (“animal welfare” OR “farm animal welfare”) OR Title/Keyword/Abstract = (“animal welfare” OR “farm animal welfare”) OR [Subject = (“pig” OR “sow” OR “boar” OR “piglet” OR “fattening pig”, “cattle” OR “cow” OR “bull” OR “beef cattle” OR “dairy cattle”, “sheep” OR “ewe” OR “ram” OR “ovis aries” OR “carpra hircus”, “chicken” OR “hen” OR “rooster” OR “laying hen” OR “broiler” OR “chick”, “duck”, “goose”, “livestock” OR “poultry” OR “livestock and poultry”) AND Subject = (“welfare” OR “animal welfare”)]}. The effectiveness of the search terms has been confirmed to a certain extent in previous studies [18,20]. Additionally, to obtain literature as widely as possible, no limitations were set for the publication year, language, or document type. The above literature search strategy was conducted through the advanced search function, and the search date was 12 June 2023. Detailed information on the literature search results can be found in the Supplementary Material (Table S1 for the non-Chinese-language literature; Table S2 for the Chinese-language literature).

### 2.2. Literature Process Strategy

To ensure the rationality and validity of the literature data, each literature record was carefully read and reviewed. Exclusion criteria used for literature selection were as follows: (1) not directly related to farm animals, such as experimental animals (mice, rats, zebrafish, etc.) and wildlife (giant pandas, elephants, tigers, etc.); (2) neither qualitative nor quantitative studies, such as research reports, government documents, conference announcements, or book reviews (for Chinese-language literature only); (3) neither a research article nor a review article, such as proceeding paper, editorial material, or book chapter (for non-Chinese-language literature only); (4) missing essential information, such as authors and institutions; and (5) duplicate literature. After several rounds of manual screening, all the valid literature data were exported in “Refworks” format and saved as plain text files (UTF-8). These files contained basic information about the literature, including the title, authors, institutions, abstract, keywords, journal, document type, published year, volume, issue, and DOI. Details of the valid literature after screening are presented in the Supplementary Material (Table S3 for the non-Chinese-language literature; Table S4 for the Chinese-language literature).

### 2.3. Literature Analysis Strategy

Bibliometric analysis is a widely used research method that can reveal the research status of a specific academic field by dealing with and analyzing published research papers using mathematical and statistical methods [29]. With the rapid development of computational and information visualization technologies, scientific knowledge mapping has become a practical bibliometric method [30]. However, there is no consensus on which bibliographic software is best [31]. CiteSpace, a Java-based data visualization analysis software developed by Professor Chen Chaomei and his team, can effectively recognize Chinese characters and thus avoid errors or unintelligible codes [21,32]. Therefore, CiteSpace (version 6.2.R2) was used to perform bibliometric analysis and create scientific knowledge maps in the present study.

CiteSpace is essentially an information visualization technique for macro knowledge measurement, which has its own unique measurement indicators and implications. It employs co-citation analysis theory, pathfinding network algorithms, minimum spanning tree algorithms, and other tools to analyze the similarity and measurement of research units (e.g., authors, institutions, and keywords) by measuring specific literature [32]. In addition to the basic statistical analysis, the specific analysis steps undertaken by CiteSpace in the current study were as follows: (1) co-authorship analysis was used to reveal the collaborative relationships among authors and institutions; (2) keyword co-occurrence analysis and keyword cluster analysis were conducted to illustrate research hotspots in the field; (3) citation burst analysis of keywords was performed to explicate research frontiers in the field.

The scientific knowledge maps created by CiteSpace do not use the original matrix consisting of the literature. Instead, they use algorithms to normalize the original matrix and then use the new matrix for network visualization. CiteSpace offers a variety of algorithms for calculating the strength of network connections, such as Cosine, Dice, and Jaccard. In the current study, the default Cosine algorithm was used [33]. The specific formula is as follows:

$$Cosine(C_{ij},\;S_{ij},\;S_{j}=\frac{C_{ij}}{\sqrt{S_{i}S_{j}}})$$

The range of Cosine is 0 to 1, where $C_{ij}$ represents the number of co-occurrences of i and j, $S_{i}$ represents the occurrence of i, and $S_{j}$ represents the occurrence of j.

The scientific knowledge maps generated by CiteSpace consist of nodes and lines. For the co-authorship analysis of authors and institutions, each node represents an author or institution, and nodes with a larger size imply that the author or institution has a larger number of publications. The lines between nodes represent collaborative relationships between two authors or institutions. Regarding the co-occurrence analysis of keywords, each node represents a keyword. The larger the node size, the more commonly the keyword appears. The lines between nodes indicate the association of keywords [34,35]. Betweenness centrality is an index that measures the importance of nodes in the network, and CiteSpace uses this index to identify and quantify the status of authors, institutions, and keywords in academic collaboration and information dissemination [36]. The calculation formula is as follows [37]:

$$Centrality({node}_{i})=\sum_{i\neq j\neq k}\frac{P_{jk}(i)}{P_{jk}}$$

Centrality ranges from 0 to 1, where $P_{jk}$ represents the number of shortest paths between nodes j and k, while $P_{jk}(i)$ indicates the number of paths that pass through ${node}_{i}$.

Regarding the cluster analysis of keywords, CiteSpace provides an automatic clustering function based on the spectral clustering algorithm. It also offers three algorithms for extracting cluster topic words from the clustering citing documents: latent semantic index, log-likelihood ratio (*LLR*), and mutual information. *LLR* was selected as the clustering algorithm in the present study, and the calculation formula is as follows [37]:

$$LLR=\log\frac{p\left(C_{j}|V_{ij}\right)}{p\left(\bar{C_{j}}|V_{ij}\right)}$$

where LLR is the log-likelihood of the keyword $W_{i}$ for category $C_{j}$, while $p\left(C_{j}|V_{ij}\right)$ and $p\left(\bar{C_{j}}|V_{ij}\right)$ are the density functions in categories $C_{j}$ and $\bar{C_{j}}$, respectively. The quality of a cluster is reflected in terms of its silhouette value, which is a measure of cluster homogeneity. Generally speaking, if the silhouette value of a certain cluster is over 0.5, the cluster obtained is considered reasonable. If the silhouette value is over 0.7, the cluster obtained is considered credible and meets the analysis requirements [35,38].

To conduct the aforementioned analysis, the plain text files of the valid literature data were imported into CiteSpace software (version 6.2.R2). The parameters for running the software for statistics and mapping were set as follows: (1) time slicing was set as 1 year per slice, covering the publication period of the literature (2002 to 2023 for non-Chinese-language literature, and 1992 to 2023 for Chinese-language literature); (2) the selection criteria for data extraction was set to the default “g-index, *k* = 25”, which means that the top 25 results were extracted for each time slice; and (3) default settings were all used for other parameters.

**Figure 1:**
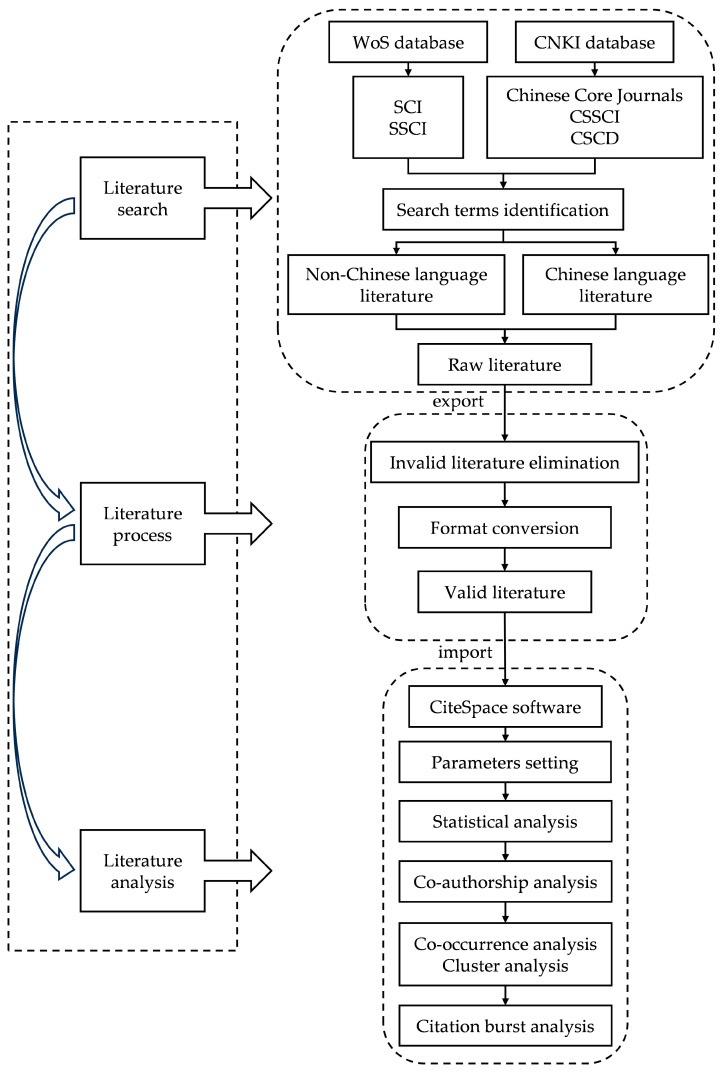
The integrated analysis framework of the study.

As an integrated analysis framework for the current study, the full strategy described above is presented in Figure 1.

## 3. Results

### 3.1. Statistical Analysis

#### 3.1.1. Annual Publication Output

A total of 5048 publication records met the search criteria, which were obtained from the WoS and CNKI databases, comprising 2128 non-Chinese-language publication records and 2920 Chinese-language publication records, respectively. After removing all ineligible papers based on the exclusion criteria, a total of 1312 Chinese farm animal welfare articles published between March 1992 and June 2023 were included and used in the final analysis and visualization, including 701 non-Chinese-language articles (from October 2002 to June 2023) and 611 Chinese-language articles (from March 1992 to June 2023). Notably, all of these 701 non-Chinese-language articles were published in English, suggesting that English is the dominant language for Chinese farm animal welfare research published in international journals.

**Figure 2:**
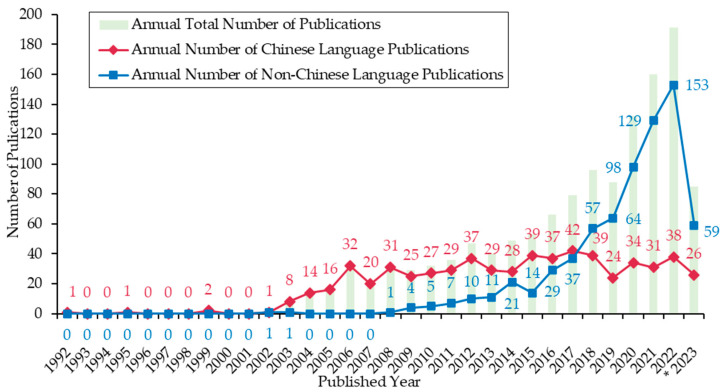
Number of articles published in the field of farm animal welfare in China from 1992 to 2023 June. * The data displayed in the figure for 2023 only includes the number of papers published during the period from January to June 2023.

The analysis temporarily excluded articles published in 2023 since the year was not yet finished, and their inclusion may not accurately reflect the overall trends. As shown in Figure 2, a total of 1227 articles were published from 1992 to 2022, averaging nearly 40 articles published per year (mean = 39.58). There was a very low level of prior output in both non-Chinese- and Chinese-language publications, with only four Chinese-language articles published before 2002 and only two non-Chinese-language articles published before 2008. A favorable and continuous fluctuating growth trend in the total number of publications starting from 2002 was observed, with an average annual growth rate of 25.60% from 2002 to 2022, indicating increasing academic attention and interest in the field of farm animal welfare in China. For non-Chinese-language publications, there has been consistent and rapid growth in annual output since 2008, with only one period of decline observed in 2015. Non-Chinese-language publications are growing at a markedly faster rate when compared to Chinese-language publications, surpassing the number of Chinese-language publications for the first time in 2018. It is worth highlighting that the number of non-Chinese-language articles published from 2020 to 2022 represented a substantial proportion (59.19%) of the total number of non-Chinese-language publications. Regarding Chinese-language publications, the annual number of Chinese-language publications has exhibited a consistent growth trend since 2002. The number of Chinese-language publications displayed large fluctuations starting in 2007, yet the average number of Chinese-language articles published per year between 2007 and 2022 consistently exceeded 31 articles (mean = 31.88).

#### 3.1.2. Distribution of Species

**Figure 3:**
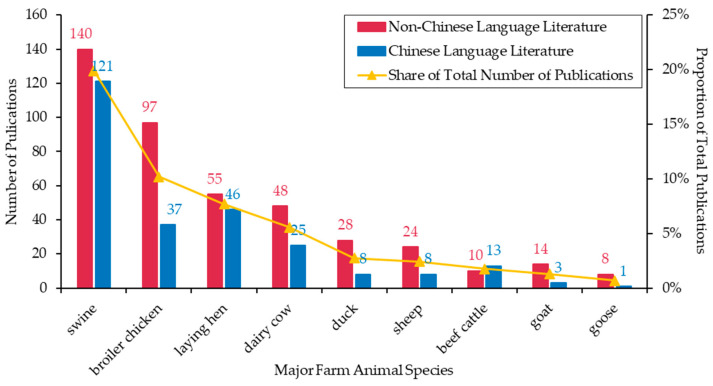
Distribution of species in the field of farm animal welfare in China.

As shown in Figure 3, the main species of interest for Chinese farm animal welfare research, ranked in descending order of priority, were swine (*n* = 261, 19.89%), chickens (*n* = 235, 17.91%), cattle (*n* = 96, 7.32%), sheep (*n* = 49, 3.73%), ducks (*n* = 36, 2.74%), and geese (*n* = 9, 0.69%). Swine are the primary focus in Chinese-language literature (*n* = 121, 19.80%) and the second focus in non-Chinese-language literature (*n* = 140, 19.97%), highlighting the important role of swine as farm animals in China. Chickens are the most commonly reported species in non-Chinese-language literature (*n* = 152, 21.68%) and the second most commonly reported species in Chinese-language literature (*n* = 83, 13.58%). There is subtle variation in the specific chicken species of interest between the non-Chinese- and Chinese-language literature. More than 60% of the non-Chinese-language literature related to chickens focused on broiler chickens (*n* = 97, 63.82%), while more than half of the Chinese-language literature related to chickens used laying hens (*n* = 46, 55.42%) as the specific species. Regarding cattle, both the non-Chinese- and Chinese-language literature demonstrate a preference for using dairy cows (*n* = 73, 5.56%) as research subjects, rather than beef cattle (*n* = 23, 1.75%). Both the non-Chinese- and Chinese-language literature exhibit a greater focus on sheep (*n* = 32, 2.44%) than goats (*n* = 17, 1.30%). Although ducks and geese received relatively little attention among poultry species, the non-Chinese-language literature showed considerably more interest in these two species than the Chinese-language literature, with nearly four times as many non-Chinese-language publications (*n* = 36, 5.14%) focusing on these species than in the Chinese-language literature (*n* = 9, 1.47%). Limited attention has been given to the welfare of other farm animal species in China, such as rabbits (*n* = 11, 0.84%), horses (*n* = 4, 0.30%), and donkeys (*n* = 4, 0.30%).

#### 3.1.3. Highly Productive Authors

Table 1 presents the top 10 most productive authors in the non-Chinese- and Chinese-language literature based on the number of publications, which was determined by CiteSpace (g-index, *k* = 25). Bao Jun, a professor at the College of Animal Science and Technology of Northeast Agricultural University and the president of ICCAW, is the most prolific author, ranking first in the non-Chinese-language literature and fourth in the Chinese-language literature. His research career focuses on investigating the behavior and welfare of livestock and poultry. Members of Bao Jun’s team, including Li Xiang, Li Jianhong, Liu Honggui, Bi Yanju, Zhang Runxiang, and Wang Chao, also achieved high rankings in the non-Chinese-language literature as co-authors of Bao Jun’s publications. Li Baoming, a professor from the College of Water Resources and Civil Engineering of China Agricultural University, emerged as the second most prolific author, ranking first place in the Chinese-language literature and fourth in the non-Chinese-language literature. Shi Zhengxiang, Zheng Weichao, Wang Chaoyuan, and Geng Ailian, who are members of Li Baoming’s team, have collaborated on numerous publications with Li Baoming, and they also hold high rankings in both the non-Chinese- and Chinese-language literature. Their main research focus is on environmental control and engineering facilities for welfare-friendly animal production. Gu Xianhong is a researcher at the Institute of Animal Science of the Chinese Academy of Agricultural Sciences that ranked third in the Chinese-language literature. Her main research area relates to the environments, stress, and welfare of livestock and poultry. Shi Binlin is a professor at the College of Animal Science at Inner Mongolia Agricultural University. He ranks fifth in the Chinese-language literature and has devoted himself to the area of nutrition and the environments of livestock and poultry. Yan Huoqi, a professor at the College of Humanities and Social Development at Nanjing Agricultural University, ranks sixth in the Chinese-language literature and has long been engaged in research on the history of animal welfare from the perspective of social sciences and the humanities. Shao Dan and Tong Haibing are researchers from a team at the Poultry Institute of the Chinese Academy of Agricultural Sciences. They are working on the nutrition and welfare of poultry, ranking seventh and eighth in the Chinese-language literature, respectively. Sun Shimin ranks ninth in the Chinese-language literature, is a professor at the College of Economics and Management of Shandong Agricultural University, and has long been concerned with farmers’ willingness and behavior to improve farm animal welfare. The centrality of the top 10 most productive authors in the non-Chinese-language literature (mean = 0.03) is slightly higher than that in the Chinese-language literature (mean = 0.00) overall, which indicates that the academic influence and collaboration of highly productive authors in the Chinese-language literature on farm animal welfare require further improvement.

#### 3.1.4. Highly Productive Institutions

The top 10 most productive institutions in the non-Chinese- and Chinese-language literature obtained using CiteSpace (g-index, *k* = 25), which ranked institutions according to their number of publications, are presented in Table 2. Highly productive institutions of farm animal welfare research in China are dominated by agricultural universities, with 10 of the 14 most productive institutions being agricultural universities. Of the other four highly productive institutions, Zhejiang University and Yangzhou University are comprehensive universities, the Chinese Academy of Social Sciences is a comprehensive research institute focusing on social sciences, and the Chinese Academy of Agricultural Sciences is an agricultural research institute. Except for the Chinese Academy of Social Sciences, which focuses on social sciences and humanities research related to farm animal welfare, the remaining 13 institutions primarily conduct research on farm animal welfare from a natural science perspective. An important commonality of these 13 institutions is the possession of a key discipline in animal science. These 14 highly productive institutions are located in North China, Central China, Northeast China, and Northwest China, where animal husbandry is relatively well developed. The vast livestock and poultry resources in these areas provided a convenient foundation for conducting farm animal welfare research. China Agricultural University and the Chinese Academy of Agricultural Sciences are in the top two positions and far ahead in terms of the number of publications and centrality, representing the high academic influence of these two institutions in the field of farm animal welfare in China. The centrality of the top 10 most productive institutions in the non-Chinese-language literature (mean = 0.12) is higher than those in the Chinese-language literature (mean = 0.07) overall, indicating that the academic influence and collaboration of highly productive institutions in the Chinese-language literature on farm animal welfare require further improvement.

#### 3.1.5. Journals-of-Choice

The top 10 non-Chinese- and Chinese-language journals, ranked by the number of publications on Chinese farm animal welfare, are listed in Table 3. The top two non-Chinese-language journals and the top two Chinese-language journals with the highest number of publications in the field of Chinese farm animal welfare each accounted for over one-fifth of the respective literature, comprising 23.82% (*n* = 167) of non-Chinese-language literature and 22.75% (*n* = 139) of Chinese-language literature. The remaining 18 journals have published less than 50 articles per journal, with a proportion of less than 10%. Nearly half of the non-Chinese-language articles (*n* = 320, 45.64%) were published in the top 10 non-Chinese-language journals with the highest number of publications in the field of Chinese farm animal welfare, while more than half of the Chinese-language articles (*n* = 331, 54.16%) were published in the top 10 Chinese-language journals by publication count, with an apparent agglomeration characteristic. The main categories of these 20 journals include veterinary science, zoology, and agricultural engineering, reflecting the multidisciplinary intersection attribution of farm animal welfare research in China. Of these 20 journals, Animals (JCR IF2022 = 3.0) has published the highest number of papers on Chinese farm animal welfare, covering a wide range of farm animal species. On the contrary, China Poultry (CNKI IF2022 = 1.066), which ranked first among Chinese-language journals in the top 10 for the highest number of publications in the field of Chinese farm animal welfare, primarily focused on poultry—especially broiler chickens and laying hens. Coincidentally, the second-ranked non-Chinese-language journal with the highest number of publications in the field of Chinese farm animal welfare, Poultry Science (JCR IF2022 = 4.4), also specializes in poultry research. Computers and Electronics in Agriculture (JCR IF2022 = 8.3), Biosystems Engineering (JCR IF2022 = 5.1), and Transactions of the Chinese Society of Agricultural Engineering (CNKI IF2022 = 3.760) are the journals among these 20 journals that focus on research related to the techniques and equipment of welfare-friendly animal production, which indirectly indicates that research on farm animal welfare in China focuses not only on theoretical innovation but also on practical applications. PLoS One (JCR IF2022 = 3.7) is the only comprehensive journal among these 20 journals. Notably, non-Chinese-language journals specifically dedicated to publishing animal welfare research, such as Animal Welfare (JCR IF2022 = 1.2, *n* = 9, 1.28%) and the Journal of Applied Animal Welfare Science (JCR IF2022 = 1.5, *n* = 3, 0.43%), do not rank among the top 10 non-Chinese-language journals with the highest number of publications in the field of Chinese farm animal welfare.

### 3.2. Co-Authorship Analysis

#### 3.2.1. Co-Authorship Analysis of Authors

**Figure 4:**
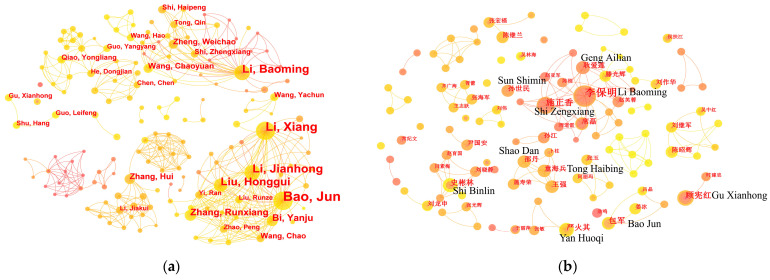
Author collaboration network map of the field of farm animal welfare in China. (**a**) Author collaboration network map of the non-Chinese-language literature; (**b**) author collaboration network map of the Chinese-language literature.

An author collaboration network map was generated using CiteSpace to illustrate collaborative relationships between the authors of non-Chinese-language articles (Figure 4a) and Chinese-language articles (Figure 4b). The current collaboration among authors in the field of farm animal welfare in China was generally characterized by overall dispersion and partial concentration. Although collaboration was the main form of publication in the field, the scope of cooperation was relatively small and fixed. Most of the research was conducted by mentors and their graduate students, who worked or studied at the same institution, while less collaborative research was performed by researchers from different institutions. According to Figure 4, two relatively large collaborative teams were observed, with Li Baoming and Bao Jun as the core of the teams. The team with Li Baoming as the core comes from China Agricultural University and seems to have the most team members while also being strongly interconnected in both the non-Chinese- and Chinese-language literature. They have demonstrated great interest in the study of environmental control and engineering facilities for welfare-friendly animal production. The team with Bao Jun as the core comes from Northeast Agricultural University and has more collaborative relationships in the non-Chinese-language literature, with relatively fewer in the Chinese-language literature. This team is dedicated to studying the behavior and welfare of livestock and poultry. In addition to these two major collaborative teams, the remaining collaborative relationships among authors involved a relatively small number of researchers, with looser interconnections to each other.

#### 3.2.2. Co-Authorship Analysis of Institutions

**Figure 5:**
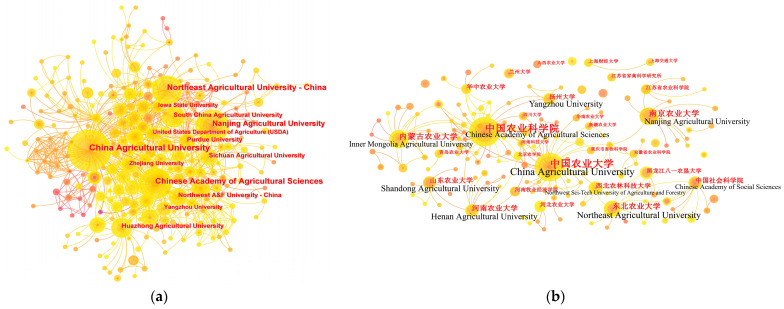
Institution collaboration network map of the field of farm animal welfare in China. (**a**) Institution collaboration network map of the non-Chinese-language literature; (**b**) Institution collaboration network map of the Chinese-language literature.

Collaborative relationships among institutions associated with the non-Chinese-language literature (Figure 5a) and Chinese-language literature (Figure 5b) were illustrated by an institution collaboration network map created using CiteSpace. Although institutes and universities were the mainstays of farm animal welfare research in China, there is no shortage of participation from livestock enterprises and government departments. This indicates that farm animal welfare has likely become a widespread problem in Chinese livestock and poultry production practices, which required the joint participation of these institutions in researching and finding solutions. This also reflects that research on farm animal welfare had important prospects for application and promotion in China. Based on Figure 5, China Agricultural University and the Chinese Academy of Agricultural Sciences could be identified as the two most influential academic institutions, whose collaborative relationships were far more than those of the others. A normal, but important fact is that most of the collaboration occurred among institutions located in the same city or neighboring cities. However, some collaboration also occurred between Chinese institutions and foreign institutions such as Purdue University, Iowa State University, and the United States Department of Agriculture.

### 3.3. Keyword Analysis

#### 3.3.1. Keywords with High Occurrence

**Figure 6:**
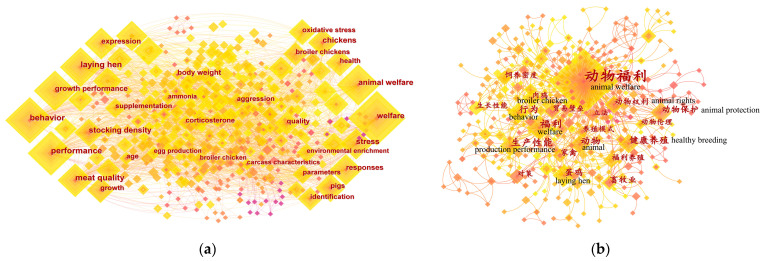
Keyword co-occurrence network map of the field of farm animal welfare in China. (**a**) Keyword co-occurrence network map of the non-Chinese-language literature; (**b**) keyword co-occurrence network map of the Chinese-language literature.

A keyword co-occurrence map of the non-Chinese-language literature (Figure 6a) and Chinese-language literature (Figure 6b) was obtained through keyword co-occurrence analysis using CiteSpace. High-occurring keywords consist of specific species and specific research topics, reflecting popular species and research hotspots in the field of farm animal welfare in China to an extent. In the non-Chinese-language literature, “welfare” is the keyword with the highest occurrence, followed by “behavior”, “performance”, “animal welfare”, and “growth performance”. In the Chinese-language literature, “animal welfare” is the keyword with the highest occurrence, followed by “welfare”, “laying hen”, “healthy breeding”, and “behavior”. Figure 6 illustrates that the keywords with high occurrence in both the non-Chinese- and Chinese-language literature share similarities in terms of species and research topics, such as “laying hen”, “welfare”, “behavior”, and “animal welfare”, indicating that there are common research hotspots in both the non-Chinese- and Chinese-language literature on farm animal welfare research in China.

To obtain a clearer picture of the specifics of the keywords, 10 keywords with the highest occurrence in the non-Chinese- and Chinese-language literature, which were counted by CiteSpace (g-index, *k* = 25), were sorted and listed in Table 4. Among these keywords, “animal welfare” had the highest centrality in both the non-Chinese- and Chinese-language literature, which illustrates that “animal welfare” occupied a key position in the farm animal welfare research in China and was closely related to other keywords. Other keywords with less centrality, including “behavior”, “performance”, “growth performance”, “meat quality”, “expression”, “responses”, “stress”, “healthy breeding”, and “production performance”, revealed the most popular research content in the field to a certain extent. Keywords related to species, including “laying hen” and “broiler chicken” reflect the most popular species in the field to some extent. Additionally, as variations or synonyms of “animal welfare” and keywords closely related to “farm animal welfare”, the centrality of keywords, including “welfare”, “animal”, “animal protection”, and “animal rights”, were also relatively low, but still larger than 0.

#### 3.3.2. Keywords Clustering Labels with High Occurrence

**Figure 7:**
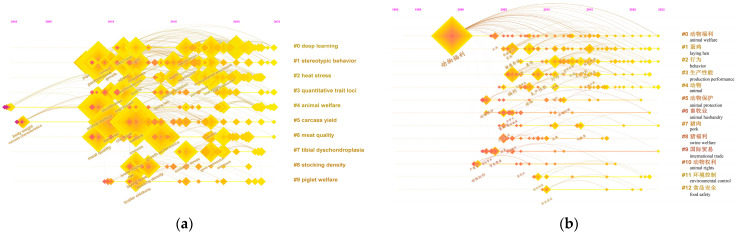
Keyword cluster map of the field of farm animal welfare in China. (**a**) Keyword cluster map of the non-Chinese-language literature; (**b**) eyword cluster map of the Chinese-language literature.

The keywords were analyzed by cluster analysis using CiteSpace, which grouped keywords from the non-Chinese-language literature (Figure 7a) and Chinese-language literature (Figure 7b) into 9 and 12 clustering labels, respectively. The sequence number of clustering labels was inversely proportional to the size of the clusters. In the non-Chinese-language literature, the largest cluster was labeled as “#0 deep learning”, followed by “#1 stereotypic behavior”, “#2 heat stress”, “#3 quantitative trait loci”, “#4 animal welfare”, “#5 carcass yield”, “#6 meat quality”, “#7 tibial dyschondroplasia”, “#8 stocking density”, and “#9 piglet welfare”. In the Chinese-language literature, the largest cluster was labeled as “#0 animal welfare”, followed by “#1 laying hen”, “#2 behavior”, “#3 production performance”, “#4 animal”, “#5 animal protection”, “#6 animal husbandry”, “#7 pork”, “#8 swine welfare”, “#9 international trade”, “#10 animal rights”, “#11 environmental control”, and “#12 food safety”.

Basic information on the top five keywords covered by clusters derived from the LLR algorithm is presented in Table 5. The silhouette values of these clusters were all larger than 0.8, indicating that the clusters were generally considered reasonable and met the analysis requirements. The specific composition of the top five clustering labels in the non-Chinese-language literature is presented as follows. The largest cluster (#0 deep learning) was formed by the keywords of 67 articles published from 2010 to 2023. The second-largest cluster (#1 stereotypic behavior) was composed of the keywords of 60 articles published between 2007 and 2023. Cluster “#2 heat stress” was the third-largest cluster, which was made up of keywords from 53 articles published from 2011 to 2022. The size of cluster “#3 quantitative trait loci” was the fourth largest and consisted of the keywords of 51 articles published from 2009 to 2023. The cluster that ranked fifth was “#4 animal welfare”, which contained the keywords of 50 articles published between 2002 and 2023. The specific composition of the top five clustering labels in the Chinese-language literature is presented as follows. The largest cluster, labeled as “#0 animal welfare”, comprised keywords from 79 articles published between 1997 and 2023. The second-largest cluster, labeled as “#1 laying hen”, consisted of keywords from 46 articles published between 2005 and 2023. The third-largest cluster, labeled as “#2 behavior”, included keywords from 42 articles published from 2007 to 2023. The fourth-largest cluster, labeled as “#3 production performance”, consisted of keywords from 39 articles published between 2005 and 2022. The fifth-largest cluster, labeled as “#4 animal”, contained keywords from 27 articles published between 2004 and 2023.

#### 3.3.3. Keywords with the Strongest Citation Bursts

The top 20 keywords with the strongest citation bursts in the non-Chinese- and Chinese-language literature are listed in Table 6, which highlights the research frontiers in the field of farm animal welfare in China. In the non-Chinese-language literature, the keywords with high burst strength, including “broiler chickens”, “performance”, and “temperature”, have been studied extensively since 2002. The keywords of “computer vision”, “recognition”, “temperature”, and “precision livestock farming” are still experiencing ongoing bursts in 2023, reflecting the present research frontiers and possible future development direction of the non-Chinese-language literature. In the Chinese-language literature, the keywords with the highest burst strength from 2003 were “behavior”, “trade barrier”, and “welfare-friendly animal production”. The keywords “laying hen” and “behavior” continued their prominence in 2023 and represent frontiers of research in recent years, potentially indicating the future development direction of Chinese-language literature.

## 4. Discussion

A comprehensive bibliometric analysis of China’s farm animal welfare research from the WoS and CNKI databases was conducted using CiteSpace software (version 6.2.R2). This bibliometric analysis statistically and visually outlines the current status, research hotspots, and research frontiers of the field of farm animal welfare in China, providing the researchers with valuable information on the general landscape and future trends in this field.

### 4.1. Number of Publications

Within this review, 1312 non-Chinese and Chinese-language publications on farm animal welfare in China published since 1992 were discovered. This validates previous findings that farm animal welfare has indeed become a research area of interest to Chinese scholars, contrary to the perception that farm animal welfare is of little interest or remains unexplored in China [14]. The results revealed that the number of publications increased from 1992 to 2022, indicating that farm animal welfare has emerged as an important topic in China, which has received increasing academic attention and achieved certain progress, despite the growing number of publications in all fields of science internationally [39]. Surprisingly, the number of articles on farm animal welfare published in China appears to surpass that of countries from Latin America. Gallo et al. [40] only collected 663 articles related to the welfare and behavior of farm animals from the WoS database and Centre for Agriculture and Bioscience Abstracts database between 1992 and 2021 in Latin America, of which 287 articles were published in Brazil and 167 articles were published in Mexico. Notably, the present study identified 489 non-Chinese-language publications related to farm animal welfare in the same period in China from the WoS database, far exceeding the number of publications on farm animal welfare in these two countries in Latin America with the highest productivity. It can be speculated that China seems highly likely to make significant progress and take a prominent position in the global field of farm animal welfare research in the near future. For example, Uyanga et al. [41] found that China has emerged as the most prolific institution in the global research on heat stress in poultry. However, research in the field of farm animal welfare in China, which requires more attention and development, remains limited when compared to that of countries in North America and the European Union [15].

The findings showed that the quantity and growth rate of non-Chinese-language literature have surpassed those of Chinese-language literature since 2018. This suggests that the recent expansion in farm animal welfare scientific output in China is primarily driven by non-Chinese-language publications in international journals, and Chinese farm animal welfare research is gaining greater visibility among readers and researchers outside of China [42]. Research has pointed out that the continuous increase in non-Chinese-language publications conducted by Chinese researchers is largely due to the policy of encouraging more international publications in Chinese universities and research institutes [43]. In spite of specific promotion criteria varying across different institutions, including monetary bonus schemes and career-related incentives, a greater emphasis has been placed on SCI and SSCI indexed journal publications than domestic publications [44]. Under such an incentive structure, rational Chinese researchers naturally tend to shift their efforts toward more valued international publishing in pursuit of considerable financial reward.

### 4.2. Development Process

Based on the changing trend of annual publication outputs (Figure 2), combined with the history of animal husbandry in China, the evolution of farm animal welfare research in China can be categorized into three specific development stages: the early stage (1992–2001), the rapid-growth stage (2002–2007), and the mature stage (2008–present).

In the early stage (1992–2001), only four Chinese-language articles were published, accounting for less than 1% of the total literature (0.30%), while no non-Chinese-language articles were published (Figure 2). The science of animal welfare originated in Western countries, and the concept of animal welfare was introduced to mainland China in the early 1990s [4]. However, in the late twentieth century, livestock and poultry production in China was mainly conducted by rural households, and intensive livestock farming was rare due to limited funding at that time [45]. Farm animal welfare, as a product of intensive livestock farming, initially received little attention from scholars in China [46].

In the rapid-growth stage (2002–2007), a total of 93 articles were published, including 91 Chinese-language articles published consistently and 2 non-Chinese-language articles published sporadically, representing 7.09% of the total literature (Figure 2). With the expansion of livestock and poultry production, intensive livestock and poultry farming was becoming increasingly prevalent in China to further improve production efficiency and economic benefits [47]. Meanwhile, since China entered the World Trade Organization in November 2001, the exportation of Chinese livestock products was widely restricted due to farm animal welfare issues, which resulted in massive economic losses [48]. Therefore, an increasing number of Chinese scholars have begun to focus on farm animal welfare issues and conduct relevant research. However, there was limited substantial progress during this period, with only a few preliminary exploratory experiments conducted, such as the influence of summer ambient temperature on the behavior of small-tailed cold sheep and their postural choices [49], the relationship between shading and environmental enrichment on feather pecking and welfare in laying hens [50], and the effect of transportation on the weight of weaned piglets [51]. The majority of studies in this period focused on qualitative analyses, such as the impact of animal welfare standards in international trade policy on China’s farm animal product exports [52,53], strategies and measures for improving farm animal welfare in livestock and poultry production [54,55], and proposals for farm animal welfare legislation [56,57].

In the mature stage (2008–present), 1215 articles were published, consisting of 516 Chinese-language articles and 699 non-Chinese-language articles, which accounted for 92.61% of the total literature (Figure 2). Currently, the priority of animal husbandry development in China has shifted from increasing the volume of livestock and poultry products to quality improvement, while the highlights of the policies have also adjusted from promoting the development of animal husbandry to emphasizing both industry development and environmental protection [58]. In this context, farm animal welfare issues caused by intensive livestock farming have received extensive attention from scholars in China, and Chinese farm animal welfare research in this stage has realized the transformation from the original ethical appeal to comprehensive scientific exploration [59]. Especially in recent years, experimental research from the natural sciences perspective and empirical research from the social sciences perspective have emerged in an endless stream. There is now a broad emerging consensus in the Chinese academic community that improving farm animal welfare has a significant promoting effect on animal husbandry quality [60,61].

### 4.3. Species Differences

According to the results, the primary focus was placed on swine and chickens within China’s farm animal welfare research, with more attention being paid to swine than chickens. This is consistent with the findings of Sinclair et al. [14], which suggested that such a focus is likely to be determined by the value of the pork industry in international trade or the perceived complexity of providing improved welfare for pigs. In contrast to the extensive research conducted on swine and poultry, there has been less farm animal welfare research focused on cattle/cows and sheep/goats in China. This diverges from the research trends observed in other countries and globally. Gautret et al. [62] found that over 30% of studies on livestock and poultry welfare and health in Europe from 2003 to 2014 concentrated on ruminants (34.62%), followed by poultry (17.40%) and pigs (13.13%). Freire and Nicol [15] also revealed that cattle/cows (13.89%) received greater research attention in global animal welfare science than pigs (13.74%) and laying hens (8.46%).

The distribution of species in Chinese farm animal welfare research may be closely related to the welfare status of farm animals in livestock and poultry production. For example, in the United Kingdom, sheep and beef cattle are typically raised using extensive management practices, and their welfare is therefore more likely to be met, with more research focused on addressing welfare concerns for pigs, poultry and dairy cattle [63]. Species distribution is also likely to be influenced by the structure of farm animal production in China. A previous bibliometric review revealed that more research on European organic livestock farming has been conducted on cattle, sheep, and poultry due to their relatively higher proportion among organic farm animals [23]. The ruminant sector, represented by beef cattle, dairy cattle, sheep, and goats, has become one of the rapidly expanding segments within China’s livestock and poultry industry, with great growth in both production and consumption of its products [1]. Nonetheless, animal welfare concerns resulting from intensive farming practices remain a major challenge to the sustainable development of the industry [64,65]. Therefore, it is necessary for Chinese researchers to conduct more studies on the welfare of ruminants in the future.

### 4.4. Major Contributors

A Matthew effect was identified among major contributors in the field of farm animal welfare in China [66]. This implies that most of the highly productive authors in the field are from the same teams which are typically led by scholars with academic influence (Table 1), and most of the highly productive institutions in the field are well-known universities or institutes in China (Table 2). This can be explained by the fact that these highly productive teams and institutions may possess stronger research resources and invest more manpower, financial, and material resources into the implementation of farm animal welfare research to increase scientific output [67,68]. This is unfavorable for newer and less established authors, but similar situations also exist in other research areas [69,70]. Moreover, the low centrality of both authors (Table 1) and institutions (Table 2), particularly in the Chinese-language literature, implies that there is still room for enhancement in terms of the collaboration among highly productive authors and institutions in the field of farm animal welfare in China, as well as the academic impact of their work [71].

Regarding journals, the agglomeration characteristic observed in Chinese farm animal welfare research may be influenced by editorial preferences and the scope of journals. As the field of farm animal welfare research expands and becomes more multidisciplinary, research on this topic may be published in a wider range of journals, potentially resulting in a weaker agglomeration characteristic. Additionally, different from global animal welfare science, non-Chinese-language journals specifically dedicated to publishing animal welfare research, such as Animal Welfare and the Journal of Applied Animal Welfare Science, are not dominate in Chinese farm animal welfare publications [15]. Although there is no Chinese-language journal specifically dedicated to publishing animal welfare research, such positive growth in China’s farm animal welfare science is likely to fuel the launch of new scientific journals that specifically focus on animal welfare.

### 4.5. Collaborative Relationships

A sound collaborative relationship among scholars can establish academic networks to share innovative ideas, concepts, and theories, generate new knowledge, and ultimately reduce the wasting of academic resources and improve the productivity of research [72]. However, it was determined that collaboration in the field of farm animal welfare in China is relatively weak among both authors (Figure 4) and institutions (Figure 5). Although the non-Chinese-language literature demonstrates a slightly higher level of collaboration when compared to the Chinese-language literature, with a greater number of collaborating authors and stronger interconnections, especially within teams, the collaborative relationships in this field could be regarded as relatively fixed on a small scale. The relatively scattered authors and institutions are unfavorable for the exchange of academic knowledge and ideas in the field, which further hinders the expansion of research content. This scenario is likely to be detrimental to the long-term sustainability of Chinese farm animal welfare research. One possible reason for this is the lack of effective academic platforms in this field, such as specialized academic conferences, which makes it difficult for scholars to communicate with and learn from each other [73].

### 4.6. Research Hotspots

Keywords are domain terms that can briefly and concisely summarize the core theme and reflect the core content of an article at a high level [74]. The results of the keywords co-occurrence analysis (Table 4 and Figure 6) are highly similar to those of international animal welfare science [15], suggesting that the research hotspots in the field of farm animal welfare in China are similar to those of international animal welfare science. This observation was also confirmed in a recent Chinese-language study in China [75]. In addition, the clustering labels of the non-Chinese-language literature tend to be more detailed and specific, while the clustering labels of the Chinese-language literature tend to be less detailed and broader (Table 5 and Figure 7). This phenomenon could be attributed to the difference in linguistic or writing conventions between English language and Chinese language [76].

Combined with a thorough review of the literature searched from databases, the results of keyword co-occurrence analysis (Table 4 and Figure 6) and keyword clustering analysis (Table 5 and Figure 7) indicate that research hotspots in the field of farm animal welfare in China can be summarized in 10 categories: “feed and diet”, “environmental impacts and control”, “integrated rearing management”, “injury and disease”, “behavior and technologies for behavior monitoring”, “genetic analysis”, “welfare during transport and slaughter”, “welfare-friendly animal product consumption”, “attitudes toward farm animal welfare”, and “healthy breeding”. As pointed out by Sinclair et al. [14], the Five Freedoms are still the central primary categories for hotspots of Chinese farm animal welfare research, although the publication of the Five Freedoms was around 30 years ago [77].

Despite the specificity of the search terms, it is noteworthy that the research hotspots identified often appear to be only peripherally related to farm animal welfare, rather than directly focused on farm animal welfare. This is mainly reflected in the large number of studies that have been conducted on the effects of factors influencing farm animal welfare on growth and production performance, as well as product quality. These factors are of economic importance, which also at times have positive impacts for farm animal welfare. Sinclair et al. [14] attributed this phenomenon to the pragmatic orientation of farm animal welfare research in China. That is, despite having the ultimate aim of improving farm animal welfare, the studies that are focused on animal husbandry practices seem to always be accompanied by the aim of increasing productivity and improving product quality. This is to be expected given the supply pressure on Chinese farm animal products. One important reason might be potential divergences in the understanding of animal welfare between China and other regions, such as European countries. This is quite understandable given the vast differences in historical and cultural backgrounds, political systems, and basic national conditions. In fact, there still remains confusion about the concepts of “animal welfare” and “animal rights” among a majority of Chinese, and welfare of animals is often wrongly construed to equate with people’s welfare in China, such as housing, nutrition, and medical care [78]. Some researchers feel compelled to avoid the term directly for fear of alienating ordinary Chinese people or the government [79]. There are various understandings of animal welfare across countries, but there is a growing consensus that farm animal welfare has to be safeguarded and enhanced.

### 4.7. Research Frontiers

According to the citation burst analysis of keywords, the current study found that “computer vision”, “recognition”, “temperature”, “precision livestock farming”, “laying hen”, and “behavior” were the keywords with the strongest citation bursts that are still experiencing ongoing bursts in 2023 (Table 6). Therefore, these keywords can be identified as research frontiers in the field of farm animal welfare in China, and future research may continue to focus on these frontiers.

With increasing capital pouring into the animal husbandry industry, the Chinese Government is encouraging and promoting the adoption of large-scale farming and modern technologies [80]. Precision livestock farming technology, which aims to achieve the real-time monitoring of farm animal health and welfare both automatically and continuously, is being widely adopted in large-scale livestock and poultry production [81]. As a result, research on precision livestock farming, such as computer vision and automatic recognition, is expected to emerge in large numbers.

Climate change is increasingly affecting livestock and poultry production with an overwhelmingly negative effect on the welfare of farm animals [82]. To avoid the serious consequences of the stress response, research on temperature control and management of farm animals in extreme weather conditions may become a potential area of future research.

Daily behavioral patterns are an essential manifestation of the health and welfare status of livestock and poultry [83]. Courses related to animal behavior, such as ethology, have been introduced in most agricultural universities in China since the early 21st century. The growing number of researchers specializing in farm animal behavior have contributed to the rapid development of farm animal behavior research in China [48].

In addition, the welfare of laying hens in China is a particular concern because some practices used for laying hens that have been phased out in other nations for animal welfare reasons remain common in China, such as non-enriched cage systems for egg production [84]. Therefore, laying hens may be a crucial species for future research.

## 5. Conclusions and Limitations

Overall, the findings of this paper are presented as follows. (1) At present, farm animal welfare research in China is relatively mature, and the scale of research is still growing. Swine and chickens have received priority attention in this area. (2) The authors and institutions in this field are scattered, with only a small number of collaborative networks formed. There also remains a lack of authors and institutions with academic influence. (3) Research hotspots in this field can be summarized in 10 categories: “feed and diet”, “environmental impacts and control”, “integrated rearing management”, “injury and disease”, “behavior and technologies for behavior monitoring”, “genetic analysis”, “welfare during transport and slaughter”, “welfare-friendly animal product consumption”, “attitudes toward farm animal welfare”, and “healthy breeding”. (4) It was determined that “computer vision”, “recognition”, “temperature”, “precision livestock farming”, “laying hen”, and “behavior” represent the major research frontiers in this field. (5) Farm animal welfare research in China is pragmatic. Enhancing growth and production performance, as well as product quality, appear to be prioritized over improving farm animal welfare.

However, the findings of this study must be understood within the confines of its limitations. First, this review applied the quantitative analysis approach only. Thus, future research can analyze China’s farm animal welfare research using both quantitative and qualitative analysis approaches. Second, the literature analyzed in this review was only obtained from peer-reviewed journals to ensure the quality of publications. Therefore, some highly relevant publications from non-peer-reviewed journals may have been overlooked, which may have resulted in an incomplete analysis. Third, the analysis conducted for the present study tended to concentrate on the top-ranking authors, institutions, journals, etc., which may have resulted in some less important literature being overlooked. However, this study provides useful baseline data that can be used to understand and track the current status and future direction in the field of farm animal welfare in China.

## Figures and Tables

**Table 1:** Top 10 most productive authors in the field of farm animal welfare in China.

Ranking	Non-Chinese-Language Literature				Chinese-Language Literature			
	Authors	Count	Centrality	Year *	Authors	Count	Centrality	Year
1	Bao J	47	0.01	2012	Li BM	17	0.00	2005
2	Li X	33	0.09	2013	Shi ZX	11	0.00	2005
3	Li JH	26	0.00	2013	Gu XH	9	0.00	1995
4	Li BM	23	0.05	2010	Bao J	8	0.00	2006
5	Liu HG	21	0.05	2014	Shi BL	7	0.00	2010
6	Bi YJ	13	0.00	2018	Yan HQ	7	0.00	2013
7	Zhang RX	13	0.00	2020	Shao D	6	0.00	2014
8	Zheng WC	8	0.00	2018	Tong HB	6	0.00	2014
9	Wang CY	8	0.08	2020	Sun SM	6	0.00	2008
10	Wang C	7	0.00	2018	Geng AL	6	0.00	2007

* The “year” in the table refers to the year when the author’s literature first appeared in the field of farm animal welfare in China, as identified by CiteSpace (g-index, *k* = 25).

**Table 2:** Top 10 most productive institutions in the field of farm animal welfare in China.

Literature Type	Ranking	Institutions	Count	Centrality	Year *
Non-Chinese-Language Literature	1	China Agricultural University	120	0.36	2003
	2	Chinese Academy of Agricultural Sciences	72	0.20	2011
	3	Northeast Agricultural University	68	0.08	2010
	4	Nanjing Agricultural University	61	0.13	2009
	5	South China Agricultural University	32	0.06	2016
	6	Huazhong Agricultural University	32	0.15	2010
	7	Northwest Sci-Tech University of Agriculture and Forestry	27	0.08	2013
	8	Sichuan Agricultural University	27	0.06	2002
	9	Zhejiang University	21	0.04	2014
	10	Yangzhou University	19	0.00	2014
Chinese-Language Literature	1	China Agricultural University	71	0.21	2005
	2	Chinese Academy of Agricultural Sciences	70	0.14	1995
	3	Nanjing Agricultural University	25	0.05	2006
	4	Inner Mongolia Agricultural University	25	0.05	2006
	5	Northeast Agricultural University	21	0.06	2005
	6	Henan Agricultural University	17	0.06	2008
	7	Shandong Agricultural University	15	0.03	2008
	8	Northwest Sci-Tech University of Agriculture and Forestry	14	0.06	2005
	9	Chinese Academy of Social Sciences	14	0.03	2003
	10	Yangzhou University	12	0.01	2009

* The “year” in the table refers to the year when the institution’s literature first appeared in the field of farm animal welfare in China, as identified by CiteSpace (g-index, *k* = 25).

**Table 3:** Journals with the top 10 highest number of publications in the field of farm animal welfare in China.

Literature Type	Ranking	Journals	Count	Proportion
Non-Chinese-Language Literature	1	Animals	110	15.69%
	2	Poultry Science	57	8.13%
	3	Computers and Electronics in Agriculture	47	6.70%
	4	Frontiers in Veterinary Science	20	2.85%
	5	Animal	18	2.57%
	6	Biosystems Engineering	18	2.57%
	7	Journal of Animal Science	14	2.00%
	8	PLoS One	13	1.85%
	9	Applied Animal Behaviour Science	12	1.71%
	10	Agriculture	11	1.57%
Chinese-Language Literature	1	China Poultry	71	11.62%
	2	Heilongjiang Animal Science and Veterinary	68	11.13%
	3	Chinese Journal of Animal Science	47	7.69%
	4	Journal of Domestic Animal Ecology	38	6.22%
	5	Transactions of the Chinese Society of Agricultural Engineering	23	3.76%
	6	Animal Husbandry and Veterinary Medicine	22	3.60%
	7	China Animal Husbandry and Veterinary Medicine	21	3.44%
	8	Chinese Journal of Animal Nutrition	15	2.45%
	9	Chinese Journal of Animal and Veterinary Sciences	14	2.29%
	10	Feed Industry Magazine	12	1.96%

**Table 4:** Keywords with the top 10 highest occurrence in the field of farm animal welfare in China.

Literature Type	Ranking	Keywords	Count	Centrality	Year *
Non-Chinese-Language Literature	1	Welfare	145	0.11	2012
	2	Behavior	99	0.12	2010
	3	Performance	98	0.17	2009
	4	Animal welfare	89	0.23	2009
	5	Growth performance	76	0.05	2014
	6	Meat quality	61	0.15	2009
	7	Laying hen	55	0.09	2012
	8	Expression	52	0.13	2011
	9	Responses	50	0.06	2012
	10	Stress	48	0.09	2011
Chinese-Language Literature	1	Animal welfare	202	0.89	2002
	2	Welfare	37	0.09	2006
	3	Laying hen	24	0.05	2008
	4	Healthy breeding	21	0.04	2006
	5	Behavior	21	0.05	2011
	6	Production performance	20	0.09	2009
	7	Animal protection	16	0.02	2003
	8	Animal	15	0.06	2008
	9	Broiler chicken	14	0.03	2013
	10	Animal rights	14	0.01	2002

* The “year” in the table refers to the year when the keyword first appeared in the field of farm animal welfare in China, as identified by CiteSpace (g-index, *k* = 25).

**Table 5:** Top five clusters of keywords in the field of farm animal welfare in China.

Literature Type	Cluster	Label (LLR, *p*-Value)	Size	Silhouette Value	Mean (Year) *
Non-Chinese-Language Literature	#0 deep learning	① Deep learning (69.47, <0.001); ② Computer vision (40.61, <0.001); ③ Precision livestock farming (18.40, <0.001); ④ Lameness detection (17.09, <0.001); ⑤ Dairy cattle (14.96, 0.001).	67	0.832	2019
	#1 stereotypic behavior	① Stereotypic behavior (14.44, 0.001); ② Physiology (14.44, 0.001); ③ Growing pigs (10.82, 0.005); ④ Enriched environment (10.82, 0.005); ⑤ Pig (9.50, 0.005).	60	0.802	2014
	#2 heat stress	① Heat stress (14.42, 0.001); ② Gut microbiota (12.49, 0.001); ③ Dairy cow (12.16, 0.001); ④ Rumen fermentation (11.51, 0.001); ⑤ Buffalo (8.10, 0.005).	53	0.814	2019
	#3 quantitative trait loci	① Quantitative trait loci (20.46, <0.001); ② Swine (15.33, <0.001); ③ Candidate gene (15.33, <0.001); ④ Single-nucleotide polymorphism (10.21, 0.005); ⑤ Perch (10.21, 0.005).	51	0.858	2014
	#4 animal welfare	① Animal welfare (29.46, <0.001); ② Willingness to pay (26.24, <0.001); ③ Choice experiment (20.97, <0.001); ④ China (11.36, 0.001); ⑤ Pork (10.46, 0.005).	50	0.824	2014
Chinese-Language Literature	#0 animal welfare	① Animal welfare (41.56, <0.001); ② Legislation (14.03, 0.001); ③ Welfare (9.04, 0.005); ④ Production performance (7.52, 0.010); ⑤ Willingness to pay (6.99, 0.010).	79	0.979	2011
	#1 laying hen	① Laying hen (26.52, <0.001); ② Welfare-friendly breeding (26.52, <0.001); ③ Healthy breeding (26.52, <0.001); ④ Poultry (17.60, <0.001); ⑤ Image processing (13.17, 0.001).	46	0.899	2015
	#2 behavior	① Behavior (37.90, <0.001); ② Growth performance (18.76, <0.001); ③ Beef cattle (11.51, 0.001); ④ Stocking density (9.73, 0.005); ⑤ Straw (9.33, 0.005).	42	0.870	2015
	#3 production performance	① Production performance (34.19, <0.001); ② Welfare (27.62, <0.001); ③ Sow (22.27, <0.001); ④ Light intensity (13.30, 0.001); ⑤ Animal welfare (13.24, 0.001).	39	0.960	2012
	#4 animal	① Animal (23.63, <0.001); ② Dairy cow (17.23, <0.001); ③ Sensor (11.44, 0.001); ④ Heat stress (7.74, 0.010); ⑤ Information monitoring (5.70, 0.050).	27	0.930	2014

* The “mean (year)” in the table refers to the average year when the keywords in the cluster first appeared in the field of farm animal welfare in China, as identified by CiteSpace (g-index, *k* = 25).

**Table 6:** Top 20 keywords with the strongest citation burst in the field of farm animal welfare in China.

Literature Type	Keywords	Year ^1^	Strength	Begin ^2^	End ^3^	Temporal Distribution ^4^
Non-Chinese-Language Literature	Meat quality	2009	3.59	2012	2015	▂▂▂▂▂▂▂ ▂▂▂ ▄ ▄ ▄ ▄ ▂▂▂▂▂▂▂▂
	Group size	2012	2.60	2012	2017	▂▂▂▂▂▂▂▂▂▂ ▄ ▄ ▄ ▄ ▄ ▄ ▂▂▂▂▂▂
	Carcass yield	2012	2.55	2012	2018	▂▂▂▂▂▂▂▂▂▂ ▄ ▄ ▄ ▄ ▄ ▄ ▄ ▂▂▂▂▂
	Stocking density	2013	4.64	2013	2015	▂▂▂▂▂▂▂▂▂▂▂ ▄ ▄ ▄ ▂▂▂▂▂▂▂▂
	Performance	2009	5.13	2016	2017	▂▂▂▂▂▂▂ ▂▂▂▂▂▂▂ ▄ ▄ ▂▂▂▂▂▂
	Intensity	2016	3.10	2016	2018	▂▂▂▂▂▂▂▂▂▂▂▂▂▂ ▄ ▄ ▄ ▂▂▂▂▂
	Identification	2010	2.60	2016	2017	▂▂▂▂▂▂▂▂ ▂▂▂▂▂▂ ▄ ▄ ▂▂▂▂▂▂
	Broiler chickens	2012	5.18	2017	2019	▂▂▂▂▂▂▂▂▂▂ ▂▂▂▂▂ ▄ ▄ ▄ ▂▂▂▂
	Systems	2017	2.62	2017	2018	▂▂▂▂▂▂▂▂▂▂▂▂▂▂▂ ▄ ▄ ▂▂▂▂▂
	Mortality	2018	3.55	2018	2020	▂▂▂▂▂▂▂▂▂▂▂▂▂▂▂▂ ▄ ▄ ▄ ▂▂▂
	Tibial dyschondroplasia	2018	3.53	2018	2019	▂▂▂▂▂▂▂▂▂▂▂▂▂▂▂▂ ▄ ▄ ▂▂▂▂
	Piglets	2008	3.23	2018	2020	▂▂▂▂▂▂ ▂▂▂▂▂▂▂▂▂▂ ▄ ▄ ▄ ▂▂▂
	Indicators	2019	2.85	2019	2020	▂▂▂▂▂▂▂▂▂▂▂▂▂▂▂▂▂ ▄ ▄ ▂▂▂
	Exposure	2019	2.85	2019	2020	▂▂▂▂▂▂▂▂▂▂▂▂▂▂▂▂▂ ▄ ▄ ▂▂▂
	Milk	2019	2.69	2019	2020	▂▂▂▂▂▂▂▂▂▂▂▂▂▂▂▂▂ ▄ ▄ ▂▂▂
	Computer vision	2020	3.69	2020	2023	▂▂▂▂▂▂▂▂▂▂▂▂▂▂▂▂▂▂ ▄ ▄ ▄ ▄
	Recognition	2020	3.69	2020	2023	▂▂▂▂▂▂▂▂▂▂▂▂▂▂▂▂▂▂ ▄ ▄ ▄ ▄
	Dairy cattle	2020	2.61	2020	2021	▂▂▂▂▂▂▂▂▂▂▂▂▂▂▂▂▂▂ ▄ ▄ ▂▂
	Temperature	2019	4.27	2021	2023	▂▂▂▂▂▂▂▂▂▂▂▂▂▂▂▂▂ ▂▂ ▄ ▄ ▄
	Precision livestock farming	2021	3.71	2021	2023	▂▂▂▂▂▂▂▂▂▂▂▂▂▂▂▂▂▂▂ ▄ ▄ ▄
Chinese-Language Literature	International trade	2003	2.19	2003	2010	▂▂▂▂▂▂▂▂▂▂▂ ▄ ▄ ▄ ▄ ▄ ▄ ▄ ▄ ▂▂▂▂▂▂▂▂▂▂▂▂▂
	Trade barrier	2004	4.13	2004	2010	▂▂▂▂▂▂▂▂▂▂▂▂ ▄ ▄ ▄ ▄ ▄ ▄ ▄ ▂▂▂▂▂▂▂▂▂▂▂▂▂
	Livestock products	2004	1.95	2004	2008	▂▂▂▂▂▂▂▂▂▂▂▂ ▄ ▄ ▄ ▄ ▄ ▂▂▂▂▂▂▂▂▂▂▂▂▂▂▂
	Countermeasure	2005	3.77	2005	2008	▂▂▂▂▂▂▂▂▂▂▂▂▂ ▄ ▄ ▄ ▄ ▂▂▂▂▂▂▂▂▂▂▂▂▂▂▂
	Animal husbandry	2005	2.30	2005	2009	▂▂▂▂▂▂▂▂▂▂▂▂▂ ▄ ▄ ▄ ▄ ▄ ▂▂▂▂▂▂▂▂▂▂▂▂▂▂
	Legislation	2004	2.14	2008	2010	▂▂▂▂▂▂▂▂▂▂▂▂ ▂▂▂▂ ▄ ▄ ▄ ▂▂▂▂▂▂▂▂▂▂▂▂▂
	Supply chain	2008	1.97	2008	2011	▂▂▂▂▂▂▂▂▂▂▂▂▂▂▂▂ ▄ ▄ ▄ ▄ ▂▂▂▂▂▂▂▂▂▂▂▂
	Animal protection	2005	2.00	2010	2011	▂▂▂▂▂▂▂▂▂▂▂▂▂ ▂▂▂▂▂ ▄ ▄ ▂▂▂▂▂▂▂▂▂▂▂▂
	Healthy breeding	2006	2.70	2012	2015	▂▂▂▂▂▂▂▂▂▂▂▂▂▂ ▂▂▂▂▂▂ ▄ ▄ ▄ ▄ ▂▂▂▂▂▂▂▂
	Broiler chicken	2013	2.43	2013	2017	▂▂▂▂▂▂▂▂▂▂▂▂▂▂▂▂▂▂▂▂▂ ▄ ▄ ▄ ▄ ▄ ▂▂▂▂▂▂
	Welfare-friendly animal production	2015	4.06	2015	2019	▂▂▂▂▂▂▂▂▂▂▂▂▂▂▂▂▂▂▂▂▂▂▂ ▄ ▄ ▄ ▄ ▄ ▂▂▂▂
	Stocking density	2015	3.65	2015	2019	▂▂▂▂▂▂▂▂▂▂▂▂▂▂▂▂▂▂▂▂▂▂▂ ▄ ▄ ▄ ▄ ▄ ▂▂▂▂
	Laying hen	2008	3.23	2015	2023	▂▂▂▂▂▂▂▂▂▂▂▂▂▂▂▂ ▂▂▂▂▂▂▂ ▄ ▄ ▄ ▄ ▄ ▄ ▄ ▄ ▄
	Animal	2008	2.62	2015	2018	▂▂▂▂▂▂▂▂▂▂▂▂▂▂▂▂ ▂▂▂▂▂▂▂ ▄ ▄ ▄ ▄ ▂▂▂▂▂
	Sow	2011	2.05	2016	2018	▂▂▂▂▂▂▂▂▂▂▂▂▂▂▂▂▂▂▂ ▂▂▂▂▂ ▄ ▄ ▄ ▂▂▂▂▂
	Influencing factor	2009	2.38	2017	2021	▂▂▂▂▂▂▂▂▂▂▂▂▂▂▂▂▂ ▂▂▂▂▂▂▂▂ ▄ ▄ ▄ ▄ ▄ ▂▂
	Production performance	2009	2.37	2017	2020	▂▂▂▂▂▂▂▂▂▂▂▂▂▂▂▂▂ ▂▂▂▂▂▂▂▂ ▄ ▄ ▄ ▄ ▂▂▂
	Behavior	2011	4.16	2018	2023	▂▂▂▂▂▂▂▂▂▂▂▂▂▂▂▂▂▂▂ ▂▂▂▂▂▂▂ ▄ ▄ ▄ ▄ ▄ ▄
	Stress	2011	2.46	2018	2019	▂▂▂▂▂▂▂▂▂▂▂▂▂▂▂▂▂▂▂ ▂▂▂▂▂▂▂ ▄ ▄ ▂▂▂▂
	Dairy cow	2011	3.72	2019	2021	▂▂▂▂▂▂▂▂▂▂▂▂▂▂▂▂▂▂▂ ▂▂▂▂▂▂▂▂ ▄ ▄ ▄ ▂▂

^1^ The “year” in the table refers to the year when the keyword first appeared in the field of farm animal welfare in China, as identified by CiteSpace (g-index, *k* = 25). ^2^ The “begin” in the table refers to the year when the keyword started to appear with high occurrence in the field of farm animal welfare in China, as identified by CiteSpace (g-index, *k* = 25). ^3^ The “end” in the table refers to the year when the keyword stopped appearing with high occurrence in the field of farm animal welfare in China, as identified by CiteSpace (g-index, *k* = 25). ^4^ The “temporal distribution” for non-Chinese-language literature spans from 2002 to 2023, and the “temporal distribution” for Chinese-language literature spans from 1992 to 2023. The light blue bar indicates that the corresponding term has not yet appeared, while the dark blue bar indicates that the corresponding term has started to appear but is being used relatively infrequently, and the red line represents the burst time when the corresponding terms appeared more prevalently during this period.

## Data Availability

All data generated or analyzed during this study are included in this published article.

## Supplementary Materials

The following supporting information can be downloaded at: https://www.mdpi.com/article/10.3390/ani13193143/s1, Table S1: Detailed information on the non-Chinese-language literature search results (WoS database); Table S2: Detailed information on the Chinese-language literature search results (CNKI database); Table S3: Detailed information on the valid non-Chinese-language literature after screening (WoS database); Table S4: Detailed information on the valid Chinese-language literature after screening (CNKI database).
